# Diverse Parents Decision-Making to Vaccinate Their Child under Five Attending Childcare Programs

**DOI:** 10.3390/jcm13082299

**Published:** 2024-04-16

**Authors:** Ernest St. John Thompson, Elizabeth Howe, Tara Kenworthy LaMarca, Ruby Natale, Jason F. Jent

**Affiliations:** Mailman Center for Child Development, Department of Pediatrics, Miller School of Medicine, University of Miami, Miami, FL 33136, USAtlk38@med.miami.edu (T.K.L.);

**Keywords:** COVID-19 vaccination, parental decision-making, children under five, childcare programs, social determinants of health, parent acculturation, migrants

## Abstract

**Background:** On 18 June 2022, Moderna and Pfizer-BioNTech COVID-19 vaccines were authorized under an Emergency Use Authorization by the United States Food and Drug Administration to prevent severe coronavirus disease in children six months to four years of age. Despite approval of the COVID-19 vaccinations for young children, there remain ongoing challenges reaching widespread coverage due to parental decision-making. Parental decision-making plays a pivotal, yet understudied, role governing vaccine adoption among this priority demographic. **Methods:** This cross-sectional analysis examined COVID-19 vaccine intentions for 320 predominately Hispanic parents of two to five-year-olds attending Miami-Dade County childcare programs in Florida USA, several months following the June 2022 emergency authorization. Parent’s self-reported survey data encompassed vaccine choices and rationales, social determinants of health, and parent immigrant status. Data analyses illustrate the associations between parent decision-making and these variables. Regression modeling and tests of independence identified predicting factors for parental vaccine decision-making. **Results:** Only 25% of parents intended to vaccinate their young child, while 34% resisted and 41% felt unsure, despite 70% personal vaccination rates. Household income under $25,000, identifying as a migrant, or testing COVID-19-positive significantly predicted unsure decision-making. The majority of hesitant groups expressed concerns around side effects (20%), safety (2.9%), and sufficiency of vaccine knowledge (3.3%). **Conclusions:** In this sample, the predominance of parents were unsure and resistant rather than accepting of having their child vaccinated despite emergency approval of the pediatric vaccine. Associations and predictive factors are examined.

## 1. Introduction

Parental decision-making to vaccinate their child, including pediatric immunizations, has been a long-standing public health priority [1,2] (Frew and Lutz, 2017; Gust et al., 2003). Vaccine decision-making in parents has been a l, long-standing concern for controlling the spread of infectious diseases. For example, a 2015 report on vaccine decision-making describes several factors related to vaccine decision-making, including understanding risks and benefits and variance between states and locales [3] (Jacobson et al., 2015). Recommendations include population health management approaches going beyond parent education, such as using every pediatric visit to vaccinate children, protocols to provide vaccinations outside of doctor visits, and stating clear recommendations [3] (Jacobson et al., 2015).

Research indicates that while child COVID-19 vaccination rates are rising, there remains wide variability between states [4] (Mbaeyi et al., 2020). The recent COVID-19 pandemic has brought childhood vaccination rates to the forefront of public health research, as parents have to decide to weigh the risks associated with a vaccine, about which little is known regarding the long-term effects on children’s health, compared to their child contracting the disease [5] (Fisher, 2022). Research on parental decision-making to vaccinate their children against COVID-19 indicates that vaccine decision-making is a public health concern. For example, in a 2022 meta-analysis, Galanis and colleagues reported that across 44 studies, 60.1% of parents indicated their intention to vaccinate their child, but with a wide range from 25.6% to 92.2% [6].

Studies from the early stages of the pandemic indicate that many parents did not vaccinate their young children. As of May 2023, only 13% of children six months to four years of age had received at least one dose of the vaccine [7] (American Academy of Pediatrics). A study of parental vaccine decision-making among children under five found that 31.3% of parents intended to vaccinate their child, while 22.6% were unsure, and 46.2% intended not to vaccinate [8] (Fisher et al., 2022). Another study, with a sample of 309 Hispanic female guardians, found that 45.6% did not intend to vaccinate their child, while 22% were unsure, and 32.4% intended to vaccinate their child [9] (Fisher et al., 2023).

The Moderna and Pfizer-BioNTech COVID-19 vaccines for children six months to four years of age were authorized under an Emergency Use Authorization by the United States Food and Drug Administration on 18 June 2022. Since the authorization, the rates of children vaccinated between the ages of six months and four years have been steadily increasing [7,10] (American Academy of Pediatrics [AAP], 2023; Centers for Disease Control and Prevention [CDC], 2024). However, although vaccination rates are increasing, they remain low [7] (AAP, 2023). As of 5 May 2023, 2.2 million children between six months and four years were vaccinated against the COVID-19 vaccine, whereas 14.9 million had yet to receive their first dose [7] (AAP, 2023). Additionally, vaccination rates vary widely across states, ranging from 3% to 45% of children having received their first dose of the vaccine [7] (AAP, 2023). Differences in demographic variables including gender, age, education level, household income, race and ethnicity, language acculturation, and time living in the United States are found to be associated with child vaccine uptake [11] (Galanis et al., 2022).

There remains a lack of clear consensus on what constitutes vaccine decision-making in parents regarding vaccinating their children. Factors that contribute to parent’s decision-making include parental concern for safety [2,12,13] (Gust et al., 2003; Kempe et al., 2020; Nyhan and Reifer, 2015), prior tendency to immunize their child [6] (Galanis et al., 2022), social determinants of health [14] (Teasdale et al., 2022), missed well-child visits [15] (Mbaeyi et al., 2020), and mistrust of medicine and governments [5,6,9] (Daniels et al., 2022; Fisher et al., 2022; Fisher et al., 2023). Parent’s knowledge and access to information also influence their decision-making regarding vaccination [16] (Bussink-Voorend et al., 2022). For example, misinformation, including through social media messaging, influences parental beliefs about the effectiveness of vaccines and children’s lack of susceptibility to infection [9,11,17] (Fisher et al., 2023; Galanis et al., 2022; Pulgaron et al., 2022). Parent’s immigration and migrant status are also contributing factors related to vaccine decision-making [6,18,19,20,21] (Daniels et al., 2022; Hajissa et al., 2022; McFadden et al., 2022; Spitzer, 2023; Tankwanchi et al., 2022). State and local governments may also influence parental decision-making. For example, Florida’s Department of Health issued a statement in March 2022 declaring that children aged five to 17 may not benefit from the vaccine if they are healthy [22] (Florida Department of Health, 2022).

On 11 May 2023, the federal government declared an end to the public health emergency for COVID-19, and the country is now shifting from a pandemic to an endemic. Vaccine uptake among children under five poses a significant challenge for controlling the spread of viruses via childcare programs. This study’s purpose is to gain an understanding of parental decision-making for vaccinating their young child. Survey data from a population of children attending childcare programs in Miami-Dade County, Florida, USA are examined to identify patterns of children’s vaccination status. Miami Dade County is majority-minority with a population that is 72.3% Hispanic [23] (American Community Survey, 2022) and is home to a large and growing immigrant/migrant population [24] (Migration Policy Institute, 2024). In the last year, Miami-Dade County saw an influx of migrant families arriving from South America and Haiti [25] (Val, 2023), and the number of children of migrant families is increasing in Miami-Dade County public schools [26] (Payne, 2023). As of 22 October 2023, only 3.9% of children between six months and four years of age in Miami-Dade County had received the COVID-19 vaccine [10] (CDC, 2022), which is lower than the national average, making understanding parental vaccine decision-making in Miami-Dade County a priority.

### The Current Study

This study uses a cross-sectional analysis of baseline data gathered from September 2022 to January 2023 from a National Institutes of Health (NIH) R01 randomized control trial being conducted in Miami-Dade County. Jump Start Plus COVID Support (JS+CS) is a virtual early childhood mental health consultation (ECMHC) model being tested with a predominately Hispanic sample of parents and children. The R01 study is specific to understanding the impact of COVID-19 on early care and education programs (for additional details, see Natale et al., 2023) [15]. 

This dataset includes information regarding parental vaccine decision-making in a primarily Hispanic sample. Understanding Hispanic parents’ vaccine decision-making is important given historical health disparities, low rates of COVID-19 vaccine uptake, and reported vaccine hesitancy in Hispanic communities [9] (Fisher et al., 2023). Additionally, the survey data include information about parents’ rationales for vaccine decision-making, their social determinants of health, and their immigration status. The data are used to answer the following research questions:

1. What is the relationship between parents’ decision-making to vaccinate their child and parents’ own uptake of the COVID-19 vaccine, and a booster dose, and parent and child COVID-19 diagnosis?
2. What factors contribute to vaccine decision-making among parents?
3. What is the relationship between parents’ decision-making to vaccinate their child, their social determinants of health, and their acculturation?
4. What factors predict parental decision-making to vaccinate their child against COVID-19?

## 2. Methods

### 2.1. Participants

The JS+CS clinical trial is currently in its second year, has been approved by the University’s Institutional Review Board (ID: 20220115), and is registered with clinicaltrials.gov. The sample consisted of parents of children between the ages of two and five attending childcare centers in Miami- Dade County within the JS+CS clinical trial (see Table 1). Of the parents in the sample (N = 320), the mean age was 34, (SD = 7.0) with the majority (91%) identifying as female and Hispanic (88%). Children’s mean age was three years and seven months (SD = 2.1). Of the parents in this sample, 70% had received at least one dose of the COVID-19 vaccine, 45% had received a booster dose, and 91% reported they had not vaccinated their child under five.

Data were collected from parents beginning in September of 2022, after the June 18th FDA emergency approval of the vaccine for children six months to four years of age, and continuing to January 2023. Data were extracted via REDCap Version 13.8.1 [27] (REDCap Consortium, Vanderbilt University, Nashville, TN, USA, 2023) data electronic capture tools hosted at the University of Miami and analyzed using SAS Studio 3.81 Enterprise Edition Software [28] (SAS Institute Inc., Cary, NC, USA, 2022).

### 2.2. Measures

Parents completed intake forms as part of the recruitment process the clinical trial. Questions from the intake survey are used in all analyses (see Table 2 for all items in the survey). Family characteristics (i.e., English proficiency, gender, race, ethnicity, country of birth, primary language spoken at home, employment, income level, and years of education) were measured using questions from the JS+CS demographic survey. Family COVID-19 precautions were measured using a dichotomized checklist of caregiver reported behaviors. The checklist follows CDC recommendations using items from the National Institutes of Health Rapid Acceleration of Diagnostics (RADx) Common Data Elements Survey [29] (NIH, Bethesda, MD, USA, 2021). For additional information see Natale et al., 2023 [15].

Parents were first categorized into three groups based on their responses to their decision-making to vaccinate their child with (1) resistant (definitely not or not too likely), (2) accepting (very likely or fairly likely) and (3) unsure (don’t know, prefer not to answer or not applicable). Parents were also asked if they had received at least one dose of the COVID-19 vaccination and if they had received a booster. Parents who did not receive at least one dose of the COVID-19 vaccine and at least one COVID-19 vaccine booster were asked to provide their reasons using preselected items. Parents were also asked a number of questions related to social determinants of health including questions about employment status, household income, education level, child health insurance status, and their immigration status. Additionally, parents were asked about their acculturation, including if they were born in the USA, their time in the USA, their English proficiency, their preferred language, and their feelings about belonging.

### 2.3. Data Analysis

Fisher’s exact tests were used to measure associations between the three levels of parent’s decision-making compared to their vaccine uptake for their self and their child, COVID-19 diagnosis, social determinants of health, and acculturation. Fisher’s exact test was originally developed to examine associations between two categorical variables in which cell sizes were less than five. It has since been expanded to include larger contingency tables where there are small cell sizes [30] (Mauger and Kauffman, 2001). Multinomial logistic regression was used to identify factors predicting parent’s uptake of the vaccine using the acceptant parents as the reference group. Factors include parent’s demographics, reasons for not getting vaccinated or a booster, social determinants of health, and their acculturation. All relationships were considered significant if the two-tailed *p*-value was 0.05 or less.

## 3. Results

### 3.1. Decision-Making to Vaccinate

Frequencies and percentages of parents’ decision-making to vaccinate their child, their own COVID-19 vaccination status, and COVID-19 test results of both parents and children are illustrated in Table 3. The Fisher’s exact test was calculated to look across the three categories of vaccination decision-making to determine if there are significant associations between these variables. The total sample size of parents was 320, of which 306 (96%) parents answered the question about vaccinating their child. Out of the 306 parents, 126 (41%) were unsure, 103 (34%) were resistant, and 77 (25%) were acceptant of the COVID-19 vaccination for their child. There were significant associations between parents’ and children’s COVID-19 diagnosis and parent decision-making to vaccinate their child. Most parents (n = 142) and children (n = 187) had never tested positive for COVID-19 and most of these same parents expressed uncertainty regarding vaccination. Out of 133 parents who tested positive for COVID-19, most were unwilling to vaccinate their children (41%), while other parents were equally unsure (29%) or willing to vaccinate their child (29%). For children with a positive COVID-19 diagnosis, the majority of parents were resistant to vaccination (39%) while 30% of parents were unsure and 31% were willing to vaccinate their child. Although most children tested negative for COVID-19, the majority of their parents were unsure about vaccinating their child. There was no significant association between parents’ COVID-19 vaccine status and their decision-making regarding vaccination for their child.

### 3.2. Reasons for Vaccine Decision-Making

Vaccine decision-making among parents without a COVID-19 vaccine. As part of the survey, parents were asked about why they chose not to get vaccinated. Survey responses allowed parents to select any and all reasons for not getting the first dose of the vaccine. Multinomial logistic regression was used to identify any significant reasons for parents’ choices. All choices were significant except for “allergic to the vaccine” and “don’t like needles”. The results are presented in Table 4.

Vaccine decision-making among parents without a booster dose. Parents who received the vaccine were asked about their reasons for not getting a booster dose. Parents could select all options that applied to them. Multinomial logistic regression was used to determine significance among parents’ selection. All response reasons were significant, and the results can be seen in Table 5.

### 3.3. Relationship between Social Determinants of Health and Decision-Making

Social determinants of health variables were included in the analysis to determine if there is a relationship with parent’s decision-making (See Table 6). Significant associations were found regarding household income and families’ migrant status. Of the 306 parents in the sample, 287 (94%) responded to the question about household income. Parents whose household income was less than $25,000 were significantly more unsure, while parents whose household income was greater than $25,000 were significantly more resistant to vaccinating their child against COVID-19. Of the 306 parents, 105 (34%) parents responded to the question about their migrant status. Of the 105 respondents who answered the question indicating that they were a migrant, most were significantly more unsure about vaccinating their child. There were no significant associations between the other social determinants of health and parents’ decision-making to vaccinate their children in terms of their race, ethnicity, employment status, child insurance status, and education.

### 3.4. Acculturation and Decision-Making

As part of the survey, parents were also asked questions about their acculturation. Significant associations were found for being born in the USA, being in the USA for greater than five years, parents’ English proficiency, and parents’ language preference (see Table 7). The only nonsignificant measure of acculturation was the parent’s sense of belonging. The majority of parents categorized as significantly more resistant to vaccinating their child were born in the United States, were proficient in English, and preferred to speak Spanish. Parents who have lived in the US for more than 5 years, are not English proficient, and prefer to speak Spanish were significantly more unsure about vaccinating their child.

### 3.5. Predictors of Parents’ Decision-Making to Vaccinate Their Child against COVID-19

Multinomial logistic regression, with acceptant parents as the reference group, was conducted to determine what significant factors predict parents’ decision-making to vaccinate their child. The models include parent’s COVID-19 diagnoses, COVID-19 vaccine status, reasons for vaccine decision-making, social determinants of health, and acculturation. Significant predictors were testing positive for COVID-19, parent income, parents’ language preference and parents’ family status (see Table 8). Parents were more likely to be in the unsure group compared to the accepting group if they tested positive for COVID-19, had a child who tested positive for COVID-19, earned less than $25,000 per year, preferred to speak English rather than Spanish, and were a migrant. Parents were also more likely to be in the unsure group than the accepting group if their family status was migrant All other variables, including parent’s demographics, parent uptake of vaccine, parent uptake of a booster dose, parent’s reasons for not getting vaccinated, family social determinants of health, and parent’s acculturation were not significant.

## 4. Discussion

This study used a cross-sectional secondary analysis of a dataset from a randomized controlled trial in Miami-Dade County, Florida, USA to gain an understanding of the COVID-19 vaccine uptake and decision-making of diverse parents of young children between the ages of two and five. This study also examined the relationship between parental decision-making to vaccinate their child, reasons behind their resistance to vaccination, their social determinants of health, and their acculturation. This study is one of the few conducted on parents of children under five. Data collection began two months after the Emergency Use Authorization of the Moderna and Pfizer-BioNTech COVID-19 vaccine for children six months to four years of age, making this one of the first reports about parents’ intentions regarding vaccinating their child. The sample of parents is primarily Hispanic, providing insight into how a high-risk population considers vaccination for their young child. In this sample, most of the families were either resistant or unsure about vaccinating their child under five regardless of if they had received the vaccine or booster themselves, tested positive for COVID-19, or if their child tested positive for COVID-19, which is a concern given families’ higher risk status.

This study is an original investigation, and, to our knowledge, the only other study exploring parents’ vaccine COVID-19 decision-making tied to sociodemographic factors, attitudes, and beliefs was a meta-analysis that included 44 studies and only some broke down responses by the age of the child [11] (Galanis et al., 2022). There have also been other investigations about child vaccine decision-making among different demographics of parents, but these studies did not focus on parents of young children under five [17] (e.g., Pulgaron et al., 2022). Studies before the Emergency Use Authorization were hypothetical in asking parents if they would get the vaccine if approved [5,11] (e.g., Fisher et al., 2022; Galanis et al., 2022). Understanding the reasons behind parental decision-making, along with associated family characteristics, particularly for very young children, is critical for driving future research and policy.

Most parents indicated they had not yet vaccinated their child for COVID-19 despite being vaccinated themselves and testing positive for COVID-19. One possibility for vaccine decision-making in children under five may be due to their young age and the perception of their vulnerability to a new, emergency-tested vaccine [31] (Kenworthy et al., 2022). In addition, the sample in this study was primarily Hispanic, which corresponds to prior research indicating a low acceptance rate for vaccination of children under five if a vaccine was approved [9] (Fisher et al., 2023). Understanding parents’ intent to vaccinate their children against COVID-19 remains a concern despite entering a post-pandemic era, given historical issues around parental reluctance to vaccinate or immunize their child [2,4,14,32] (Gust et al., 2004; Mbaeyi et al., 2020; Shui et al., 2006; Teasdale et al., 2022).

Parent assignment to a decision-making category was significantly associated with COVID-19 diagnosis, household income, and migrant status. Significant predictors of parent decision-making category assignment were parent beliefs, COVID-19 diagnosis, parents’ preference for speaking English, and their migrant status. As previous studies have identified, there are both perceived benefits and risks associated with vaccine efficacy and safety [2,33] (Gust et al., 2004; Song et al., 2014), including intrapersonal and interpersonal beliefs that are consistent with these findings [8] (Corben and Leask, 2016). Our findings about the association between household income level and language acculturation and parent’s decision-making to vaccinate their children are similar to other studies on parental vaccine hesitancy for childhood immunizations generally [2,14,21,32,34] (Gust et al., 2003; Shui et al., 2006; Nuzhath et al., 2020; Tankwanchi et al., 2021; Teasdale et al., 2021) and to vaccination against COVID-19 specifically [5,9] (Fisher et al., 2022; Fisher et al., 2023). Other studies have also found that parents who are immigrants or migrants are more likely to be unwilling to vaccinate their children, and migrant populations tend to be under-immunized and at greater risk for contracting vaccine-preventable viruses [6] (Daniels et al., 2022). Barriers for vaccination in migrant populations include gaps in knowledge, access to healthcare, and mistrust [6] (Daniels et al., 2022). Immigrants are an under-immunized population even before COVID-19 [6] (Daniels et al., 2022). Yet, by contrast, in our sample, those who resided in the US for a longer period were more hesitant about getting their child vaccinated. This contrast makes time in the US an important area for future research. Vaccine decision-making among parents, particularly in diverse, low-income minority populations, poses a potential barrier to achieving widespread immunization coverage, especially for children under five years old.

### Implications for Future Research and Policy

In this study, parents with higher income, greater language acculturation, and lower education were less likely to vaccinate their children. Studies of children under five have varying results regarding factors underlying parental decision-making. Additional research on parental decision-making for vaccinating their children under five is needed, including factors related to their social determinants of health. Moreover, future research can clarify the variability between parents to support the development and implementation of public health campaigns that can guide policymakers in identifying which populations require the most information. 

This study also found that parents who identified as migrants were more unsure about vaccinating their children under five. This finding is consistent with other research on vaccine hesitancy in general, and vaccine hesitancy for COVID-19 specifically, in migrant and immigrant populations [9,11] (Fisher et al., 2023; Galanis et al., 2022). It is crucial to continue to gauge vaccine decision-making in migrant parents to identify root causes of hesitancy and determine improved communication strategies to increase vaccine acceptance. A focus on migrant and immigrant families is particularly vital, as migrant families have disproportionately higher rates of COVID-19 infection [35] (Balakrishnan, 2021), potentially due to occupations as front-line workers with more exposure to viruses [36] (Fabreau et al., 2022).

Policymakers also need to consider public health campaigns to address parental decision-making to vaccinate their child using the parental reasons identified in this study as a guide for messaging. Addressing parent beliefs is necessary for enacting behavior change. However, it is important to understand what prior research indicates about communication with parents around childhood vaccines and immunization, given that vaccine decision-making is a complex issue [11] (Galanis et al., 2022). For example, only providing information on vaccine safety and effectiveness to hesitant parents can be deleterious [13] (Nyhan and Reifer, 2015). Public health communication strategies and materials for parental decision-making regarding child vaccination should balance information to include both the risks and benefits [37,38] (Dubé et al., 2015; Dubé et al., 2020). A widespread public health campaign should consist of various media outlets and the use of healthcare workers, public health and community leaders, and social influencers [39] (Schaffer DeRoo et al., 2020). Additionally, government and health agencies should rely on theoretical models, such as behavioral change theories, to guide the implementation of public health campaigns [40,41] (Lin et al., 2020; Opel et al., 2009).

## 5. Limitations

This study captures a period shortly after the Emergency Use Authorization of the Moderna and Pfizer-BioNTech COVID-19 vaccines. Factors contributing to parental decision-making to vaccinate their child may have changed over time. Difficulty in running the Fisher’s exact test using the three vaccine decision-making categories (acceptant, resistant, and unsure) and its association with the multiple categories of the household income and parents’ education level impacted our ability to group parents by more nuanced social determinants of health categories. In the analysis, more than 20% of the cells across the three categories of parent vaccine decision-making for household income and parent education level had an expected count of less than five, which rendered the test invalid because there were so many categories. We collapsed some of the cells due to their small size, preventing us from understanding a broader range of household income and parent education levels. In addition, we were not able to look across both race and ethnicity due to participants who responded as being both white and black. This is consistent with a small body of literature identifying cultural differences in the interpretation of what ethnicity and race mean, in addition to concerns about limiting ethnicity to Hispanic versus non-Hispanic which fails to capture other cultural variations. Finally, the small sample of acceptance parents (25%) reduces the validity of the finding. We considered combining the resistant and unsure groups; however, prior literature [11] (e.g., Galanis et al., 2022) consistently reports parental decision-making using these three categories. We opted to use the three categories, so this research is comparable to prior studies, to improve understanding about parental decision-making for vaccinating their child.

## 6. Conclusions

To our knowledge this study is one of the first to capture data on parental decision-making to vaccinate their child under five following the Emergency Use Authorization of the Moderna and Pfizer-BioNTech COVID-19 vaccine for children six months to four years of age. The findings can impact what we know about vaccines for young children in general and guide emergency responses for future pandemics. Our study has illuminated the complex interplay of beliefs, social determinants of health, and acculturation levels influencing parental willingness to vaccinate their child under the age of five. Despite the clear risks posed by the disease, the decision is far from straight forward. Our findings underscore the significant role parental beliefs play in shaping health decisions. This study highlights the necessity of culturally sensitive public health strategies that acknowledge and address the diverse background and beliefs or our sample population. In addressing the challenges identified in this study, it is critical for public health officials and policymakers to collaborate with community leaders, healthcare providers, and educators to foster an environment of trust and support around vaccination. By understanding and addressing the nuanced barriers to vaccination faced by parent of young children, we can move closer to achieving higher vaccination rates in this age group, mitigating the impact of COVID-19. This study contributes to the research by examining the associations between parent’s beliefs, their social determinants of health, and acculturation and how they contribute to their decision-making. Vaccines are vital to stop the spread of infectious diseases such as COVID-19, making parental vaccine decision-making for their young children a public health priority to prevent future disease outbreaks.

## Figures and Tables

**Table 1:** Demographics of parents.

Demographic Variable		N (%)
Race	N = 311	
	White	258 (83)
	Black	35 (11)
	Multiracial	10 (3)
	Native American	3 (1)
Ethnicity	N = 314	
	Hispanic	276 (88)
	Non-Hispanic	32 (11)
	Haitian	5 (1)
Gender	N = 309	
	Female	282 (91)
	Male	27 (9)
Age	N = 320	
Mean (SD)	3.7 (2.1)	33 (7)

Note: 5 participants reported being unsure for race and 1 unsure for ethnicity.

**Table 2:** Measure Items.

Construct	Intake Form Question	Response Options
Parental decision-making to vaccinate their child against COVID-19	How likely are you to accept an approved COVID-19 vaccine for your child now that it is available?	Scale: very likely, fairly likely, not too likely, definitely not, don’t know, prefer not to answer, and not applicable
COVID-19 Vaccine Uptake	Have you received at least one dose of the COVID-19 vaccine?	No, Yes, Don’t know, Refused
	Have you received at least one COVID-19 booster?	No, Yes, Don’t know, Refused
Parent COVID-19 diagnosis	Have you ever tested positive for COVID-19 (using a rapid point-of-care test, self-test, or laboratory test) or been told by a doctor or other healthcare provider that you have COVID-19?	No, Yes, Don’t know, Refused
Child COVID-19 diagnosis	Has your child ever tested positive for COVID-19 (using a rapid point-of-care test, self-test, or laboratory test) or been told by a doctor or other healthcare provider that you have COVID-19?	No, Yes, Don’t know, Refused
Parents reasons for not getting the COVID-19 vaccine (check all that apply)	Why have you NOT received a COVID-19 vaccine?	allergic, don’t like needles, not concerned about getting sick, concerned about side effects, don’t believe the pandemic is bad, don’t want to pay, don’t know enough vaccine works, other
Parents reasons for not getting the COVID-19 booster (check all that apply)	Which of the following, if any, are reasons that you have not received a COVID-19 booster dose	not eligible, plan to but no appointment or no time, don’t believe it is necessary, my doctor hasn’t recommended it, already had COVID-19, not required to by work or school, experienced side effects from previous dose, not transportation, other
Parent’s social determinants of health		
	Employment status	Employed, Unemployed, Disabled, Retired, Other
	Annual household income *	Less than $25,000, $25,00–34,999, $35,000–49,999, $50,000–74,999, $75,000–99,999, $100,000–149,999, $150,000 or more
	Child has health insurance	Yes, No
	Parent’s education *	Elementary or less, Some high school, High school/GED, Technical training, Some college, Associate degree, Bachelor’s degree, Graduate degree
	Family status	(circle one) Military, Migrant, Dependency, Delinquency, N/A
Acculturation		
	How long have you lived in the United States?	Open ended years and months
	Do you feel at home in the United States?	Yes, No
	What is your primary language?	English, Spanish, Creole
	How well do you speak English?	Scale: Very well, Well, Not well, Not at all
	What language do you prefer?	English, Spanish, Creole

Note: * Response items collapsed to account for small cell size.

**Table 3:** Parent’s decision-making to vaccinate their children.

	Sample *N*	Resistant *n* (%)	Unsure *n* (%)	Acceptant *n* (%)	*p*-Value
Total number of parents	N = 306	n = 103 (34)	n = 126 (41)	n = 77 (25)	
Parent test results (COVID-19 diagnosis)					0.0017 **
Positive	133	55 (41)	39 (29)	39 (29)	
Negative	142	40 (28)	74 (52)	28 (20)	
Child test results (COVID-19 diagnosis)					0.0083 *
Positive	100	39 (39)	30 (30)	31 (31)	
Negative	187	61 (33)	83 (44)	41 (23)	
Parent Vaccine Status (1st dose)					0.1306
Received 1st dose	204	67 (33)	82 (40)	55 (27)	
Declined 1st dose	69	30 (44)	25 (36)	14 (20)	
Parent Vaccine Status (booster)					0.6367
Received booster dose	92	29 (32)	39 (42)	24 (26)	
Declined booster dose	101	32 (32)	32 (31)	29 (29)	

Note: * *p*-value < 0.05, ** *p*-value < 0.001.

**Table 4:** Reasons for vaccine decision-making among parents who did not receive the COVID-19 vaccine.

Parent Responses (*n* = 302)	Vaccination Decision-Making	
	OR (95% CI)	*p*-Value
I’m not concerned about getting really sick from COVID-19	0.060 (0.012–0.297)	0.006 *
I’m concerned about side effects from the vaccine	0.020 (0.006–0.071)	<0.0001 **
I don’t think vaccines work very well	7.965 (0.861–73.862)	0.0675
I don’t trust that the vaccine will be safe	0.029 (0.006–0.134)	<0.0001 **
I don’t believe the COVID-19 pandemic is as bad as some people say	<0.001 (<0.001–0.009)	<0.0001 **
I don’t want to pay for it	375.86 (0.20–>999.9)	0.1232
I don’t know enough about how well a COVID-19 vaccine works	0.033 (0.013–0.086)	<0.0001 **

Note: * *p*-value < 0.05, ** *p*-value < 0.0001.

**Table 5:** Reasons for COVID-19 vaccine decision-making among parents who did not receive a booster dose.

Parent Responses (*n* = 211)	Vaccine Decision-Making	
	OR (95% CI)	*p*-Value
I am not yet eligible to receive a COVID-19 booster dose	0.006 (<0.001–0.044)	<0.0001 ***
I plan to get a booster and am eligible, but haven’t made an appointment or haven’t had time to do it	0.015 (0.03–0.068)	<0.0001 ***
I don’t believe a COVID-19 booster is necessary	0.027 (0.009–0.077)	<0.0001 ***
My doctor has not recommended it	0.040 (0.004–0.358)	0.0041 *
I already had COVID-19	0.083 (0.020–0.345)	0.0006 **
I am not required to get a COVID-19 booster (by my work or school)	0.033 (0.006–0.195)	0.0002 **
I experienced side effects from my previous dose(s) of the COVID-19 vaccine	0.047 (0.005–0.420)	0.0061 *
It’s hard for me to get a COVID-19 booster dose because I do not have transportation or cannot get an appointment	0.013 (<0.001-0.463)	0.0174 *

Note: * *p*-value < 0.05, ** *p*-value < 0.001, *** *p*-value < 0.0001.

**Table 6:** Relationship between parental decision-making to vaccinate their child and their social determinants of health.

			Parental Decision-Making to Vaccinate Their Child			
		Sample *N*	Resistant *n* (%)	Acceptant *n* (%)	Unsure *n* (%)	*p*-Value
Race		304				0.095
	Black		14 (41)	11 (32)	9 (26)	
	Multiracial		7 (70)	1 (10)	2 (20)	
	Native American		0	0	3 (100)	
	White		79 (31)	64 (25)	109 (43)	
Ethnicity		287				0.788
	Haitian		3 (60)	1 (20)	1 (20)	
	Hispanic		87 (32)	67 (25)	114 (43)	
	Non-Hispanic Black		6 (40)	5 (33)	4 (27)	
	Non-Hispanic White		6 (35)	4 (24)	7 (41)	
Employment Status		284				0.572
	Disabled		0	0	1 (100)	
	Employed		84 (36)	58 (25)	93 (40)	
	Other		4 (40)	3 (30)	3 (30)	
	Unemployed		14 (25)	16 (28)	27 (47)	
Annual Household Income		287				<0.0001 *
	<$25 k		34 (27)	24 (19)	67 (54)	
	$25 k or more		69 (32)	53 (43)	40 (25)	
Child has insurance		280				0.753
	No		6 (27)	7 (32)	9 (41)	
	Yes		93 (34)	70 (26)	110 (40)	
Education		106				0.789
	No college		24 (37)	7 (32)	9 (41)	
	Some college or higher		18 (43)	70 (26)	110 (40)	
Family status		280				<0.0001 *
	Military		0	1 (100)	0	
	Migrant		18 (17)	38 (36)	49 (47)	
	Dependency		5 (38)	0	8 (62)	
	Not Applicable		68 (42)	32 (20)	61 (38)	

Note: * *p*-value < 0.0001.

**Table 7:** Fisher’s exact test of parents willing to vaccinate their children across indices of acculturation.

Acculturation	Sample *N*	Resistant *n* (%)	Acceptant *n* (%)	Unsure *n* (%)	*p*-Value
Born in USA	56				0.0015 **
		30 (54)	7 (13)	19 (34)	
Time in USA	287				0.0138 *
	Less than 5 years	90 (40)	54 (24)	83 (37)	
	5 or more 5 years	13 (20)	23 (36)	29 (45)	
English proficiency	191				<0.0001 ***
	Yes	55 (52)	25 (24)	25 (24)	
	No	19 (22)	30 (35)	37 (43)	
Language preference	284				0.0002 **
	Spanish	54 (29)	51 (27)	82 (44)	
	English	47 (51)	24 (26)	22 (24)	
	Creole	0	1 (100)	0	
	Other	1 (33)	0	2 (67)	
Sense of belonging	300				0.1012
	Yes	101 (34)	75 (25)	118 (39)	
	No	0	1 (0.03)	5 (2)	

Note: * *p*-value < 0.05, ** *p*-value < 0.001, *** *p*-value < 0.0001.

**Table 8:** Predictors of COVID-19 vaccination decision-making of parents to vaccinate their children.

	Unsure vs. Acceptant		Resistant vs. Acceptant	
	OR (95% CI)	*p*-Value	OR (95% CI)	*p*-Value
Child tested positive	0.510 (0.262–0.994)	0.05 *	0.779 (0.407–1.491)	0.45
Parent tested positive	0.464 (0.244–0.893)	0.02 *	1.061 (0.549–2.054)	0.860
Parent income <$25,000 year	7.124 (1.851–27.415)	0.004 **	2.086 (0.569–7.641)	0.267
Parent prefers to speak English	0.164 (0.032–0.838)	0.03 *	0.414 (0.092–1.855)	0.249
Migrant parents	0.194 (0.036–1.039)	0.055 *	0.047 (0.007–0.301)	0.0013 **

Note: * *p*-value < 0.05, ** *p*-value < 0.001.

## Data Availability

The data presented in this study are available on request from the corresponding author. The data are not publicly available due to privacy restrictions.
